# Prediction of breath‐holding spells based on electrocardiographic parameters using machine‐learning model

**DOI:** 10.1111/anec.13093

**Published:** 2023-11-07

**Authors:** Mohammad Reza Khalilian, Saeed Tofighi, Elham Zohur Attar, Ali Nikkhah, Mahmoud Hajipour, Mohammad Ghazavi, Sahar Samimi

**Affiliations:** ^1^ Department of Pediatrics, School of Medicine Shahid Beheshti University of Medical Sciences Tehran Iran; ^2^ Department of Cardiology, School of Medicine Tehran University of Medical Sciences Tehran Iran; ^3^ Department of Pediatrics, Mofid Children Hospital Shahid Beheshti University of Medical Sciences Tehran Iran; ^4^ Mofid Children Hospital Shahid Beheshti University of Medical Sciences Tehran Iran; ^5^ Hepatology and Nutrition Research Center, Institute for Children's Health Shahid Beheshti University of Medical Sciences Tehran Iran; ^6^ Department of Pediatrics, School of Medicine Kashan University of Medical Sciences and Health Services Kashan Iran

**Keywords:** breath holdinig, electrocardiography, machine learning

## Abstract

**Background:**

Breath‐holding spells (BHS) are common in infancy and early childhood and may appear like seizures. Factors such as autonomic dysfunction and iron deficiency anemia are thought to contribute to the incidence of BHS. In this study, electrocardiographic (ECG) parameters of patients with BHS were compared to those of healthy, normal children. Logistic regression and machine‐learning (ML) models were then created to predict these spells based on ECG characteristics.

**Methods:**

In this case–control study, 52 BHS children have included as the case and 150 healthy children as the control group. ECG was taken from all children along with clinical examinations. Multivariate logistic regression model was used to predict BHS occurrence based on ECG parameters. ML model was trained and validated using the Gradient‐Boosting algorithm, in the R programming language.

**Results:**

In BHS and control groups, the average age was 11.90 ± 6.63 and 11.33 ± 6.17 months, respectively (*p* = .58). Mean heart rate, PR interval, and QRS interval on ECGs did not differ significantly between the two groups. BHS patients had significantly higher QTc, QTd, TpTe, and TpTe/QT (all *p*‐values < .001). Evaluation of the ML model for prediction of BHS, fitting on the testing data showed AUC, specificity, and sensitivity of 0.94, 0.90, and 0.94 respectively.

**Conclusion:**

There are repolarization changes in patients with BHS, as the QTc, QTd, TpTe, and TpTe/QT ratio were significantly higher in these patients, which might be noticeable for future arrhythmia occurrence. In this regard, we developed a successful ML model to predict the possibility of BHS in suspected subjects.

## Graphical abstract

In this case–control study, we initially compared different electrocardiographic (ECG) characteristics of children with a history of the breath‐holding spell (BHS) versus normal subjects, then we developed and evaluated a machine‐learning model to predict BHSs in children with such suspected attacks, based on their ECG parameters.

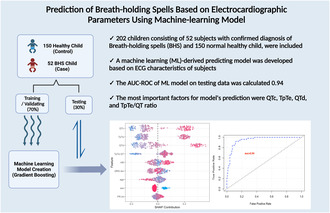

## 1 INTRODUCTION

Breath‐Holding spells (BHS) are recognized as paroxysmal non‐epileptic events, which are seen in infants and young children, mostly between 6 months to 5 years of age (Breningstall, 1996; DiMario, 2001; Leung et al., 2019). It occurs in about 0.1–4.6% of otherwise healthy children (Esmaeili et al., 2019). The exact mechanism of BHS is not known well, but autonomic dysregulation, increased vagal tone, delayed myelination of the brain stem, and also iron deficiency anemia at these ages are considered potential causes (Anil et al., 2005; Leung et al., 2019; Mocan et al., 1999; Tomoum et al., 2018). Typically, BHS disappeared until the age of six, as after this age, the autonomic system of the child has become matured (Akpinar et al., 2019). The autonomic dysfunction could lead to severe bradycardia, ventricular asystole, and cerebral hypoxia, which eventually presented as these attacks. The spells always occur in awake times, start with a crying period, and are followed by prolonged expiration. Regarding skin color during attacks, BHSs are usually divided into two categories: pallid spells and cyanotic spells; the cyanotic spells are the more common type (Amoozgar et al., 2013; Laxdal et al., 1969).

Evaluation of BHS patients' electrocardiographic (ECG) features has revealed remarkable changes, particularly in the repolarization period (Akpinar et al., 2019). Some studies have shown increased QT dispersion in these patients compared to the normal population (Akalin et al., 2004; Yilmaz et al., 2014). In addition to the QT interval, other ECG parameters of the repolarization period including T‐peak to T‐end (TpTe) and T‐peak to T‐end dispersion (TpTe dis) have been shown to have a significant correlation with future arrhythmogenic events (Castro Hevia et al., 2006; Karadeniz, 2020), and one study recently showed these parameters are significantly increased among BHS individuals (Akpinar et al., 2019).

This study aimed to investigate the differences in ECG parameters between BHS cases and normal control populations, and then, to develop a machine‐learning‐based model to predict future breath‐holding spells based on ECG parameters.

## 2 METHODS

### 2.1 Study design and patient enrollment

This case–control study enrolled a total of 202 subjects including 52 patients (25.7%) with an established diagnosis of BHS (cases), and 150 (74.3%) normal children without any history of BHS (controls). All of the subjects were collected from neurology and cardiology clinics of Mofid Children's Hospital, Tehran, Iran; from January 2020 to January 2021. All children with at least one breath‐holding spell (including all forms of it), confirmed by a pediatric neurologist or cardiologist were enrolled in the study and included in the case group; while the subjects in the control group were selected randomly from children who attended their routine annual check‐ups and were in good health during the examinations. They did not have any clinical issues at the time of the check‐up. Exclusion criteria were the presence of any underlying cardiac disease or neurologic disorder, electrolyte imbalance, hypoglycemia, renal failure, consumption of any drugs for BHSs, or suspected BHS diagnosis.

All electrocardiographic parameters were measured by a pediatric cardiologist on a 12‐lead ECG at rest, obtained using a cardiocare electrocardiography machine. QT interval measured from QRS initiation to the end of the T wave. PR interval was measured from the initial of the P wave to the peak of the R wave. On a surface ECG, the length of the QT interval corresponds to the duration of time required for all ventricular depolarization and repolarization events to take place. Heart rate is one of the most important parameters that affect the QT interval (Funck‐Brentano & Jaillon, 1993), so we used the corrected QT interval (QTc) instead, to obviate the effect of heart rate. QTc interval was calculated using the Bazet formula (QTc = QT/√RR). QT dispersion (QTd) was determined as the difference between the largest and the shortest QTc. T‐peak to T‐end (TpTe) was calculated from the peak of the T wave to the end of it, where the wave returns to the isoelectric line. Eventually, the TpTe/QT ratio was also calculated using the TpTe and the QT measurements.

### 2.2 Statistical analysis

Continuous quantitative variables are presented as mean and standard deviation, and the normality of distribution was calculated using the Kolmogrov–Smirnov test. Qualitative variables are illustrated as frequency percent. A comparison of quantitative and qualitative variables between the two groups was performed with the Student's *T*‐test and the Chi‐Square test. A multivariable logistic regression model was created to predict the breath‐holding spells regarding all ECG parameters, and its performance results were compared with the machine learning model. All of the statistical analysis was done using the SPSS software (IBM, version 26). Statistical significance was determined as a *p*‐value of less than .05.

### 2.3 Logistic regression model

Logistic regression has been the most commonly used model for the estimation of an outcome variable in medical research projects when the outcome is categorical. Logistic Regression estimates the likelihood of a single outcome from a pair of potential outcomes (binary regression). This task is done by employing the logarithm of the odds (log‐odds), and the prediction of the target variable's class (in our investigation, BHS not happening versus BHS happening) is determined according to this probability (LaValley, 2008). Eventually, we could evaluate the algorithm prediction function by fitting it to our data, determine its performance metrics, and compare them to our machine learning model performance.

### 2.4 Machine‐learning model development

**FIGURE 1:**
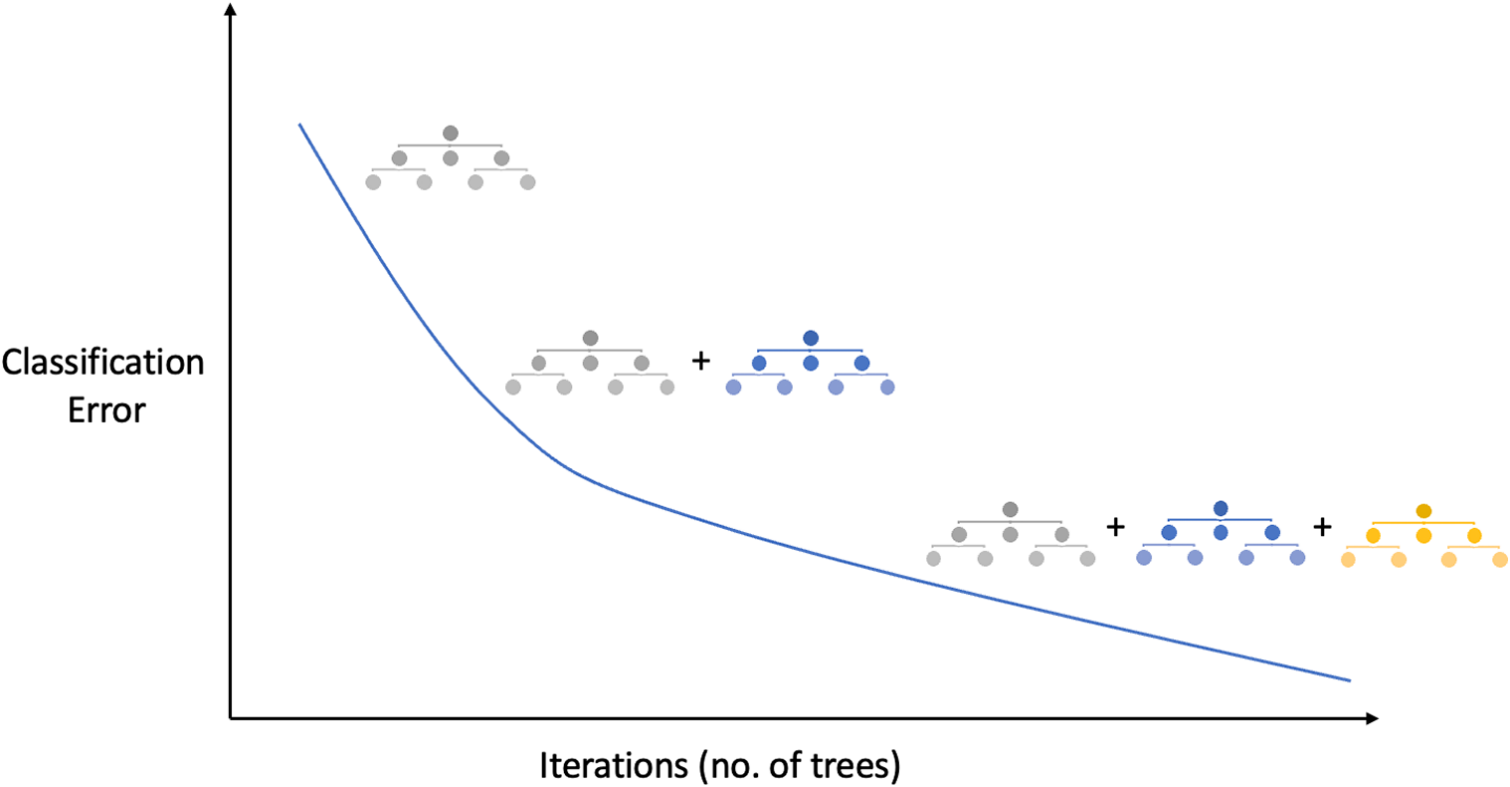
The plot shows how an ideal Gradient Boosting model works, illustrating a gradual reduction in classification error with the inclusion of one decision tree in each iteration. The final model leverages the collective strength of all decision trees to make predictions.

To develop a model to predict future breath‐holding spells based on ECG parameters, we used a supervised machine‐learning method, named the “Gradient Boosting” (GB) model. For categorization issues, one popular machine‐learning technique is the GB model. As a member of the ensemble method class, gradient boosting combines several models (referred to as “weak learners”) to enhance their performance and create the best possible final model (Figure 1).

Equation (1) describes how a simple machine‐learning model works (Zhang et al., 2019). This model tries to estimate a target variable (*y*) using a prediction function (*F*) and a set of independent features (*X*), but there is always an error (*e*), which is the difference between the predicted and the original value of target variable.

(1) $$y_{i}=F_{1}\left(X_{i}\right)+e_{i}$$

If this model is considered a weak learner, then there might be a correlation between *E* and *y*, which could be trained like Equation (2).

(2) $$e_{i}=E\left(X_{i}\right)+e_{i}^{\prime }$$

where *e*′_*i*_ is noise and has not any correlation with *y*. So, we can update Equation (1), as given below.

(3) $$F_{2}\left(X_{i}\right)=F_{1}\left(X_{i}\right)+E\left(X_{i}\right)$$

If we perform this task sequentially for *N* steps, Equation (4) would be the ultimate algorithm that the gradient boosting model utilizes to make its predictions.

(4) $$F_{N}=F_{N-1}\left(X_{i}\right)+E_{N-1}\left(X_{i}\right)$$

Therefore, the GB model iteratively leverages the errors from previous steps to enhance its performance and accuracy. The decision tree model is typically the core of the weak learners. The GB model has recently become increasingly popular in analyzing healthcare data due to its excellent prediction accuracy compared to other machine‐learning or conventional statistical methods.

The whole dataset (*n* = 202) was divided into three categories: 1. Training set (*n* = 106), 2. Validation set (*n* = 35), and 3. Testing set (*n* = 61). The training and the validation sets were used for the initial training and cross‐validation of the model during the learning process, respectively. After this, the testing set (its data were not seen by the system during learning) was used to evaluate the prediction performance of the final model.

Development of this model and further evaluations were done using the R programming language in the RStudio software environment (version 22), using the H_2_O machine‐learning package and the grid‐search method.

## 3 RESULTS

A total of 202 subjects consisting of 52 patients with a confirmed history of BHS and 150 healthy children (control group) were included in the study. The distribution of genders was statistically similar in each group, such that 37 (71.2%) and 110 (73.3%) were males for BHS and control groups, respectively (*p* = .857). Also, both groups were of the same age, and the mean age in BHS and control groups were 11.90 ± 6.63 and 11.33 ± 6.17 months old, respectively (*p* = .58). Electrocardiogram characteristics including the mean of heart rate, PR‐interval, and QRS segment did not show a significant difference between the two groups. However, all electrocardiogram parts related to the repolarization period, including QTc, QTd, TpTe, and TpTe/QT, were significantly higher in BHS patients compared to normal healthy subjects (all with *p* < .001) (Table 1).

**TABLE 1:** Comparison of the ECG parameters between the two study groups.

Characteristic	Control (*n* = 150)	Case (*n* = 52)	*p*‐Value
Age	11.90 ± 6.63	11.33 ± 6.17	.58
HR	124.53 ± 7.71	124.44 ± 8.01	.94
PR interval	115.23 ± 5.19	114.71 ± 5.46	.53
QRS duration	55.03 ± 5.00	54.90 ± 5.73	.87
QTc	404.64 ± 14.39	417.13 ± 10.58	<.001
QTd	28.87 ± 3.96	33.79 ± 3.39	<.001
TpTe	54.60 ± 4.23	60.29 ± 4.24	<.001
TpTe/QT	0.13 ± 0.01	0.14 ± 0.01	<.001

*Note*: Values are displayed as mean ± standard deviation.

Abbreviations: HR, Heart rate; QTc, Corrected QT; QTd, QT dispersion; TpTe, Inteval of peak T wave to the end of T wave.

**FIGURE 2:**
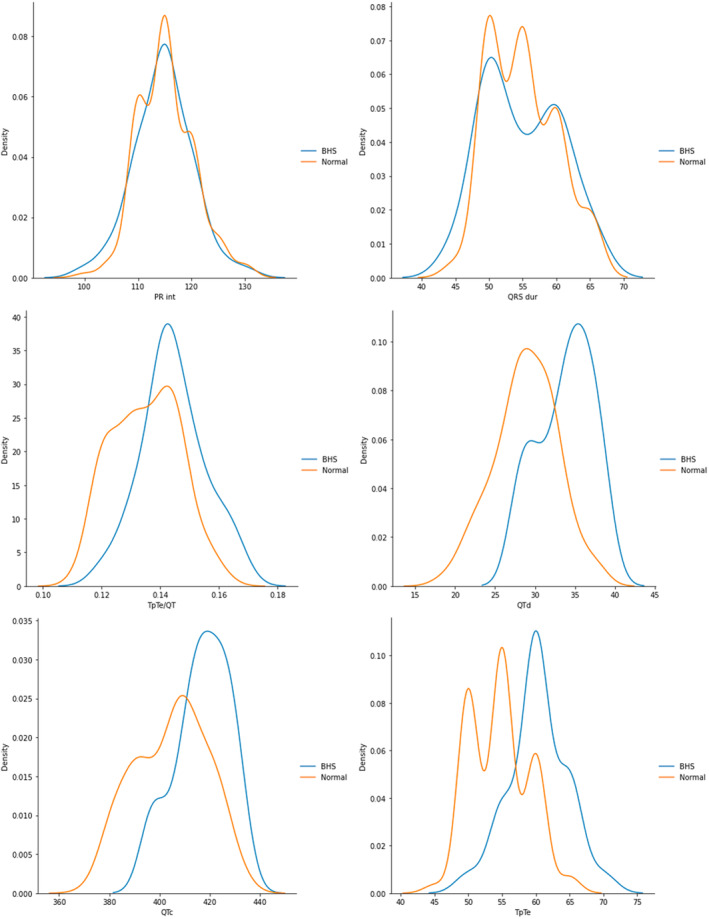
The Kernel Density Estimate (KDE) plots to compare the distribution of Gaussian density of different ECG parameters in BHS and normal children. PR int, PR interval; QRS dur, QRS duration; QTc, Corrected QT; QTd, QT dispersion; TpTe, Interval from the peak of T wave to the end of T wave.

The Kernel Density Estimate plot (KDE plot) is an alternative method to the histogram, demonstrating the distribution of Gaussian density in the dataset regarding all features (Ghosh et al., 2006). As shown in Figure 2, four ECG parameters including TpTe/QT, QTc, QTd, and TpTe have remarkably different distributions between BHS and normal healthy groups, while other parameters such as PR interval and QRS duration have approximately similar distributions between the two groups. As we can see, these plots also highlight the same results of the univariate analysis as illustrated in Table 1.

We also performed a subgroup analysis of BHS patients regarding their attack types including cyanotic or pallor. Table 2 demonstrates the comparison of ECG parameters between these two types. There aren't any remarkable differences in PR, QRS, and QTc intervals, however, repolarization variables including QTd, TpTe, and TpTe/QT ratio are significantly higher among the pallid compared to the cyanotic type BHS patients.

**TABLE 2:** Comparison of the ECG parameters between Cyanotic versus Pallid type spells.

Characteristic	Cyanotic (*n* = 48)	Pallid (*n* = 4)	*p*‐Value
HR	124.3 ± 7.9	125.7 ± 10.2	.73
PR interval	114.6 ± 5.5	115.0 ± 4.0	.91
QRS duration	55.1 ± 5.7	52.5 ± 5.0	.38
QTc	416.6 ± 10.7	423.2 ± 6.9	.23
QTd	33.5 ± 3.3	36.7 ± 2.2	.05
TpTe	59.7 ± 3.8	66.2 ± 4.7	.003
TpTe/QT	0.14 ± 0.01	0.15 ± 0.01	.01

*Note*: Values are displayed as mean ± standard deviation.

Abbreviations: HR, Heart rate; QTc, Corrected QT; QTd, QT dispersion; TpTe, Inteval of peak T wave to the end of T wave.

A multivariable logistic regression model was used to predict spells with variables including age, sex, and all ECG features. Table 3 shows the details of each variable in the model, highlighting that the most important variable for the logistic regression model is QTc (*p* < .001).

**TABLE 3:** Logistic regression model.

Characteristic	*B*	SE	Exp(*B*)	95% CI for Exp(*B*)	*p*‐Value
Sex (male)	0.30	0.56	1.36	0.44–4.12	.56
Age	−0.04	0.04	0.95	0.87–1.05	.37
HR	0.01	0.03	1.01	0.95–1.08	.67
PR interval	−0.03	0.05	0.96	0.86–1.07	.48
QRS duration	−0.005	0.04	0.99	0.90–1.09	.91
QTc	0.11	0.19	1.12	0.60–1.32	.57
QTd	0.36	0.07	1.44	1.24–1.67	<.001
TpTe	1.52	1.37	4.59	0.30–68.46	.26
TpTe/QT	0.19	0.37	1.21	0.76–3.67	.36

Abbreviations: HR, Heart rate; QTc, Corrected QT; QTd, QT dispersion; SE, Standard error of estimates; TpTe, Inteval of peak T wave to the end of T wave.

### 3.1 Machine learning model versus logistic regression model

**FIGURE 3:**
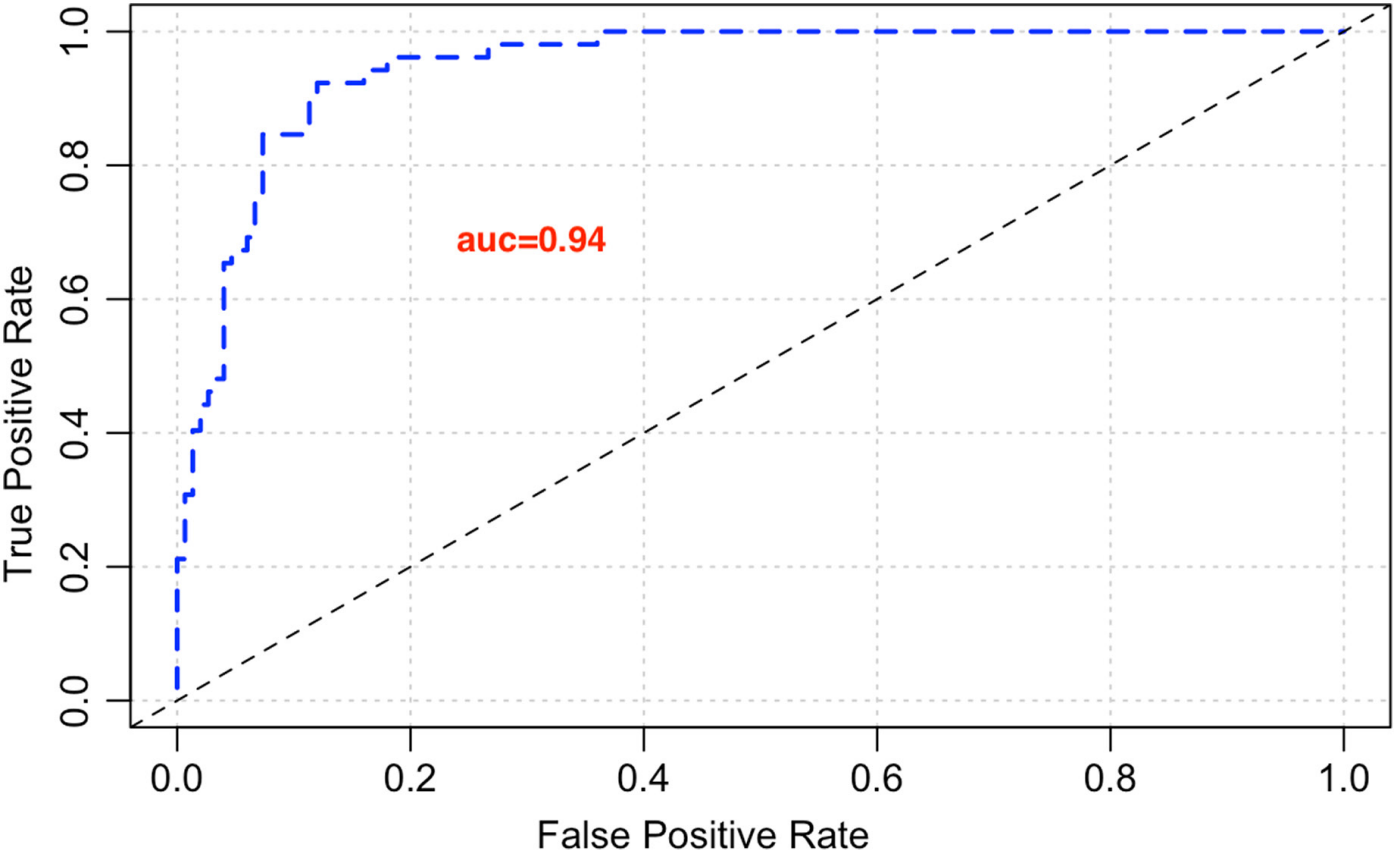
The receiver operating characteristic curve (ROC‐curve) of the machine learning model after fitting it on the testing dataset. AUC, area under the curve.

To compare our two models, we employed standard parameters commonly used to assess the performance of prediction models. These parameters include AUC, sensitivity, specificity, precision, recall, and the F‐1 score. Table 4 depicts the performance metrics of the ML model versus the logistic regression (LR) model for the prediction of BHS occurrence regarding ECG parameters. The AUCs of the ROC plot (AUC‐ROC) for the ML and LR models are 0.94 versus 0.81, respectively (Figure 3), with sensitivity and specificity of 0.94 and 0.90 for the ML and 0.69 and 0.94 for the LR models. Furthermore, the overall accuracy of the ML model for predicting breath‐holding spells based on ECG parameters is 0.91, while the accuracy of the LR model is 0.87; also, the F1 score that indicates the harmonic mean of the precision and recall indices of a classifier algorithm equals 0.87 and 0.74 respectively for ML and LR models. Accordingly, we reached the conclusion that the ML model (Gradient Boosting method) is superior to the LR model in terms of the BHS prediction function.

**TABLE 4:** Machine learning (Gradient boosting) and Logistic regression models performance metrics.

ML model	F1 score	Accuracy	Sensitivity	Specificity	Recall	Precision	AUC‐ROC
Gradient boosting	0.87	0.91	0.94	0.90	0.94	0.81	0.94
Logistic regression	0.74	0.87	0.69	0.94	0.69	0.80	0.81

Abbreviations: AUC‐ROC, the area under the curve of the Receiver Operating Characteristic plot; ML, Machine learning.

### 3.2 Feature importance

**FIGURE 4:**
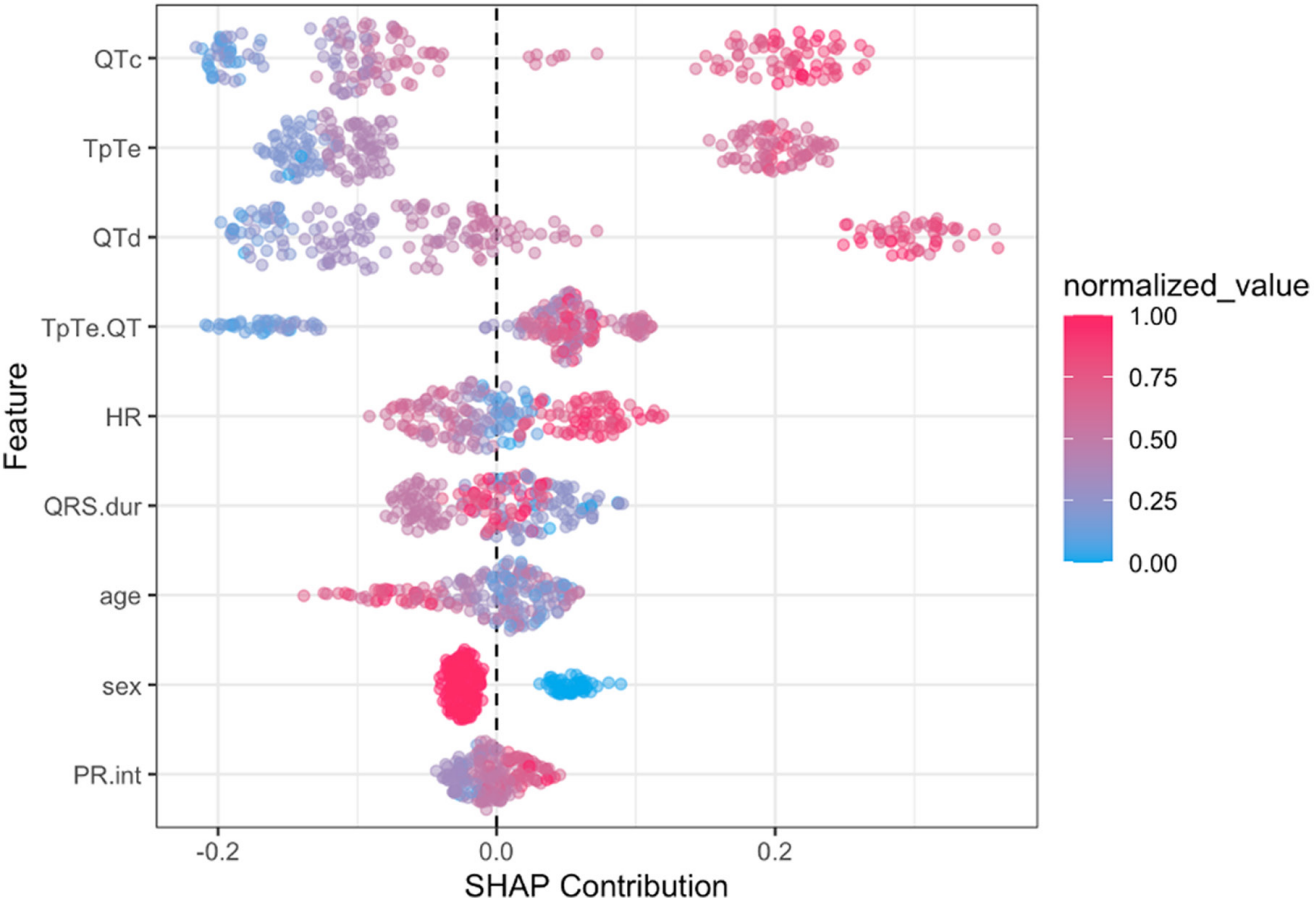
SHAP summary plot, representing all features used for system prediction, sorted by their impact on the decision‐making process of the machine learning algorithm. The red and blue dots show each feature's direct and indirect impact on the final prediction, respectively. HR, heart rate; PR int, PR interval; QRS dur, QRS duration; QTc, Corrected QT; QTd, QT dispersion; TpTe, Interval from the peak of T wave to the end of T wave.

The previous metrics are essential for the evaluation of the model performance, but after the development of a verified and efficient model, we should determine the importance of each feature in the final prediction. SHAP summary plot is a method that helps us to make this task interpretable so that we can easily explain our model (Lubo‐Robles et al., 2020). As shown in Figure 4, which is the SHAP summary plot of the ML model, the features are listed downward (on the *y*‐axis) in order of their impact on the system decision making, and the *x*‐axis is demonstrating their SHAP contribution for distinguishing the two groups. So, the most striking result to emerge from the SHAP plot of our model is that QTc, TpTe, QTd, and TpTe/QT, respectively, are the most powerful features the algorithm utilizes to predict its final results.

## 4 DISCUSSION

When dealing with high‐dimensional correlations between features, machine‐learning methods usually performs more efficiently than conventional methods in generating predictive models for medical datasets. In this study, we developed a highly efficient machine‐learning model named Gradient Boosting model, to predict future breath‐holding spells based on ECG parameters, with an AUC of 0.94 and an overall accuracy of 0.91. Moreover, we used the multivariable logistic regression model on our data to predict the BHS attacks. A comparison of these 2 models revealed that the ML model is superior to the LR model respecting its better overall performance metrics.

In this investigation, we demonstrated that the mean values of various repolarization ECG parameters, such as corrected QT, QT dispersion, TpTe, and the TpTe/QT ratio, are notably higher in the BHS group compared to the group of normal, healthy children. Conversely, other parameters like heart rate, PR interval, and QRS duration did not exhibit any statistically significant differences between the two groups. Some studies have previously reported a correlation between QT dispersion (QTd), corrected QT dispersion (QTcd), and TpTe measurements with ventricular conduction disruptions, abnormal repolarization, and the occurrence of ventricular arrhythmias. (Castro‐Torres, 2014; Jaromin et al., 2021; Kors et al., 2008; Wang et al., 2007). Akalin et al. studied 43 subjects with BHS and compared their ECG parameters with the control group consisting of 25 normal children, and concluded that QT interval and QTc had not a remarkable difference, while QT dispersion (QTd) and QTc dispersion (QTcd) were significantly higher in BHS compared to control group (59.5 ± 35.9 ms and 44.8 ± 11.9 ms, for QTd and 102.1 ± 41.9 ms and 79.6 ± 24.6 ms for QTcd, respectively, in BHS and control groups, *p* < .01 for both) (Akalin et al., 2004). In another study by Amoozgar et al., among QT dispersion, QTc dispersion, TpTe dispersion, and P wave dispersion, only QTc dispersion was significantly higher in the BHS group rather than the normal group (148.2 ± 33.1 ms vs. 132 ± 27.3 ms, *p* = .01) (Amoozgar et al., 2013). Movahedian et al. evaluated 12‐lead ECG of 56 BHS individuals and compared them with the normal healthy group. There was a significant difference between the patient and control groups in terms of QTd and QTcd (*p* < .001) (Movahedian et al., 2016). Kolkiran et al. investigated ECG parameters during and after spells in BHS patients (37 BHS cases including 30 cyanotic and 7 pallid) and realized that respiratory sinus arrhythmia and asystole periods are more common during breath‐holding spells, especially in pallid types, while there was no significant difference between BHS patients with the normal control group (26 healthy children) in terms of repolarization ECG parameters (including QT, QTc, QTd, QTcd). They eventually confirmed the presence of autonomic dysregulation in subjects with breath‐holding spells (Kolkiran et al., 2005).

In a retrospective cohort study by Cosgun and colleagues, they surveyed 124 diabetic patients within two groups: no diabetic retinopathy (NDR) and diabetic retinopathy (DR), and demographic data along with ECG parameters were compared between the 2 groups. Among all features, TpTe and TpTe/QT were remarkably prolonged in the DR group compared to the NDR group. Furthermore, the multivariate logistic regression analysis revealed that TpTe (OR = 6.01, %95 CI = 4.17–7.52, *p* = .012), TpTe/QT (OR = 1.215, 95% CI = 0.874–1.612, *p* = .029), and diabetes mellitus duration (OR = 1.422, 95% CI = 1.146–1.712, *p* < .001) were independent variables to predict DR occurrence. Eventually, they concluded that diabetic patients with concomitant retinopathy might be at a higher risk of sudden cardiac death because of increased ventricular arrhythmias risk in this group (COŞGUN et al., 2021).

In terms of the spell types, we concluded that QTd, TpTe, and TpTe/QT ratio were significantly higher among the pallid type group rather than patients with the cyanotic spells. Our results are consistent with most previous studies in the literature. DiMario et al. compared these BHS types regarding their QTc and concluded that QTc is remarkably higher in pallid‐type spells (DiMario, 2004). Furthermore, Akalin and colleagues showed that the QTd and also QTcd are significantly higher in pallid and mixed‐type patients compared to cyanotic‐type subjects (Akalin et al., 2004).

This study underscored the significance of ventricular repolarization abnormalities in individuals experiencing breath‐holding attacks. Ventricular repolarization constitutes a critical phase in cardiac electrical activity, and even minor irregularities within this phase could pose a substantial risk for potentially fatal ventricular arrhythmias. The modulation of ventricular repolarization closely correlates with the autonomic nervous system function. As a child matures and the autonomic nervous system becomes more developed, the occurrence of repolarization disruptions decreases, leading to an improvement in breath‐holding spells.

BHS is typically considered a benign transient condition in childhood; Nevertheless, according to evidence from this study and former similar articles, the presence of noticeable repolarization disturbances in this particular group of children that could put the patients at a greater risk of lethal arrhythmias should be emphasized more in clinical practice and future research in this field.

Our study has some limitations. First, this survey was conducted as a single‐center case–control study involving a limited population. Increasing the size of our case group, comprised of BHS patients, would likely enhance the algorithm's learning curve, thereby resulting in greater precision and improved final prediction accuracy. Second, the current study exclusively focused on analyzing ECG features among BHS patients, overlooking other potential factors that could contribute to predicting breath‐holding spells in children. For instance, considering variables like iron‐deficiency anemia in conjunction with ECG data could enhance the predictive capabilities of the model. The third limitation pertains to the absence of external validation for our developed model. While our model exhibited strong performance on our testing dataset, its robustness could be further established through validation on external, standard data obtained from a different medical center. This external validation would enhance the generalizability of the model to a broader population of BHS patients.

## 5 CONCLUSION

To the best of our knowledge, this is the first study for the development of a machine‐learning model to predict the possibility of breath‐holding spell occurrence in suspected children regarding their baseline ECG parameters. Based on our model, the most important features to distinguish BHS from the normal groups respectively were QTc, TpTe, QTd, and TpTe/QT. Further prospective works need to be carried out to confirm the results of our algorithm.

## AUTHOR CONTRIBUTIONS

Conception and design: Tofighi S. Administrative support: Khalilian MR. Provision of study materials or patients: Nikkhah A and Attar EZ. Collection and assembly of data: Hajipour M and Ghazavi M. Data analysis and interpretation: Tofighi S. Manuscript writing: Tofighi S and Samimi S.

## FUNDING INFORMATION

None.

## CONFLICT OF INTEREST STATEMENT

The authors of this study have no conflict of interest to declare.

## ETHICS STATEMENT

This study was conducted in accordance with the declaration of Helsinki, and approval from the Research Ethics Committee of School of Medicine – Shahid Beheshti University of Medical Sciences. Ethics board approval ID: IR.SBMU.MSP.REC.1401.383.

## PATIENT CONSENT STATEMENT

All patients were informed of the study protocol and the publication of its results. Written informed consent was obtained from all participants.

## Data Availability

The data supporting this study's findings are available from the corresponding author upon reasonable request.
